# Polymorphisms of the sodium voltage-gated channel, alpha subunit 1 *(SCN1A -A3184G)* gene among children with non-lesional epilepsy: a case-control study

**DOI:** 10.1186/s13052-022-01350-2

**Published:** 2022-09-02

**Authors:** Esraa Ghazala, Doaa A. Shahin, Yahya Wahba

**Affiliations:** 1 Department of Pediatrics, Mansoura University Faculty of Medicine, Mansoura, Egypt; 2 Department of Clinical Pathology (Hematology), Mansoura University Faculty of Medicine, Mansoura, Egypt

**Keywords:** Children, Epilepsy, PCR, *SCN1A* gene

## Abstract

**Background:**

Mutations in the neuronal sodium voltage-gated channel, alpha subunit 1 (*SCN1A)* gene have been associated with epilepsy. We investigated the *SCN1A-A3184G* polymorphism among Egyptian children and adolescents with non-lesional epilepsy.

**Methods:**

A prospective case – control observational study was done in Mansoura University Children’s Hospital, Egypt including 326 children with non-lesional epilepsy (163 antiepileptic drugs (AEDs) resistant cases & 163 AEDs responders) and 163 healthy controls. One step real time polymerase chain reaction (PCR) was used for the molecular analysis. Student’s t-test, and Monto Carlo, chi-square and Mann–Whitney tests were used for the statistical analysis.

**Results:**

All study participants were matched as regards the age, sex and body weight (*p* = 0.07, 0.347 and 0.462, respectively). They had the (AA) and (AG) genotypes but not the (GG) variant. No significant differences were found between cases and controls regarding (AG) and (AA) genotypes and A- and G-alleles (*p* = 0.09 and 0.3, respectively). We did not find significant differences between AEDs responders and resistant cases regarding the studied genotypes and alleles (*p* = 0.61 and 0.746, respectively). In the resistant group, we observed significant associations between the (AG) genotype and seizure frequency (*p* = 0.05), the tonic-clonic seizure (*p* < 0.001), the younger age of first seizure attack (*p* = 0.03), abnormal electroencephalogram (EEG) (*p* < 0.001), the positive family history of epilepsy (*p* = 0.006), topiramate (*p* = 0.03) and valproic acid (*p* < 0.001), while the (AA) genotype was associated with carbamazepine (*p* = 0.03). While in AEDs responders, there were significant associations between the AG genotype and the abnormal EEG activity, levetiracetam and carbamazepine (*p* = 0.016, 0.028 and 0.02).

**Conclusions:**

The *SCN1A-A3184G* genotypes and alleles were not associated with the epilepsy risk among Egyptian children. Significant associations were reported between the AG genotype and some predictors of refractory epilepsy.

## Background

Epilepsy is a multifactorial channelopathy disease with the involvement of both acquired and genetic factors [1]. Knowing the exact etiology of epilepsy is crucial for patients’ treatment and for neurobiological researchs that could direct future personalized therapies [2]. Neuronal voltage-gated sodium channels (SCN) are proteins responsible for the generation and propagation of the action potentials within the neurons. This occurs through affection of the membrane permeability to sodium ions and facilitation of the ions diffusion down an electrochemical gradient till the sodium equilibrium potential [3].

There are evidences about the role of the *SCN* polymorphisms in the epilepsy pathogenesis. These polymorphisms have been associated with a spectrum of epilepsy syndromes such as generalized epilepsy with febrile seizures plus, borderline severe myoclonic epilepsy of infancy, Dravet syndrome, Doose syndrome and infantile spasms [4, 5]. Most of *SCN* mutations are within *SCN1A* gene and fewer are within the *SCN2A*, *SCN8A* and *SCN1B* genes [6]. The *SCN1A* gene is located in the chromosome 2 (2q24.3) and includes 26 exons. *SCN1A* encodes the alpha subunit of the sodium channel NaV1.1 [7]. This type of channels conduct sodium ions through pores in the cellular membranes. Its architecture comprises four domains with six segments; four homologous domains (D1–D4), each containing six α-helical segments (S1–S6). The positively charged residues in the S4 segment or voltage-sensing helix of each domain are involved in the the graded membrane potential changes and generation of the action potentials [8].

Several studies have reported the efficacy of the sodium-channel blockers for the treatment of epilepsies due to genetic channelopathies. Carbamazepine, oxcarbazepine, phenytoin, lamotrigine, lacosamide and lidocaine prevent seizure activity through blocking the movement of sodium ions across sodium ion channels during the propagation of action potentials. This group of antiepileptic drugs (AEDs) has an additional advantage through acting on potassium channels, due to structural similarities, thus controlling genetic epilepsies due to mutations of voltage-gated potassium channel genes [9].

The *SCN1A-A3184G (p.Thr1067Ala)* polymorphism has been suggested to be involved in the gating of sodium channels, thus rendering them insensitive to sodium-channel blockers [10, 11]. The association of the *SCN1A-A3184G* polymorphism with the epilepsy risk was investigated in several non-Caucasian populations [12–15] and in a limited number of Caucasian populations [10, 16] with inconsistent results, thus necessitating additional studies.

Some studies reported significant associations between the *SCN1A* polymorphisms and AEDs resistance [8]. Others tried to investigate the possible associations between these polymorphisms and AEDs choice and doses. Tate et al. reported a significant association between the *SCN1A* polymorphism and the dose of phenytoin and carbamazepine, and suggested a trend of reduction in the maximum dose required according to the genotype [17]. Heinzen et al. noted that individuals with the AA genotype of the *rs3812718* variant need higher doses of AEDs than others with the GG genotype [18].

In the current research, we investigated the patterns and frequencies of *SCN1A-A3184G (p.Thr1067Ala)* polymorphism among Egyptian children and adolescents with non-lesional epilepsy, including both AEDs responders and resistant cases. We tested the association between *SCN1A-A3184G* genotypes and some predictors of refractory epilepsy. We also highlighted the possible pharmacological implication of studying the target polymorphism, through describing the frequently used AEDs and their possible link with the genotypes.

## Methods

### Study design and participants

We conducted a preliminary prospective case – control study in Mansoura University Childrens’s Hospital, Mansoura, Egypt from February 2020 to January 2022. We enrolled 326 children with non-lesional epilepsy; 163 AEDs resistant patients and 163 AEDs responders. We included 163 healthy children of comparable genders and ages as a control group. We recruited the controls from the same hospital while they attended for regular follow-up visits or for minor complaints such as pharyngitis and mild gastroenteritis. All controls had no history of neurological disorders, and were of the same ethnic origin as the patients.

Non-lesional epilepsy was described if there was a history of at least two unprovoked seizures accompanied by epileptiform electroencephalogram (EEG) changes in a patient with normal neurological examination and development, and no structural lesions detectable by magnetic resonance imaging [19]. AEDs resistance was defined if at least four seizure attacks happened over 1 year after using the maximum tolerated doses of three appropriate AEDs [20, 21]. Recovery from fits for at least 1 year after starting AEDs was the hallmark for considering the child as an AEDs responder [20, 22]. Patients with a single episode of seizure, metabolic derangement, secondary epilepsies and poor compliance to therapy were excluded.

### Clinical data collection

We retrieved relevant data of the patients from archive files including their gender, age, the seizure type and frequency, the age of onset of seizures, the duration since the last seizure attack (in months), EEG findings and AEDs therapies. We assessed all patients for the seizure type (tonic, tonic-clonic, clonic, myoclonic and absence) using the 2017 International League Against Epilepsy (ILAE) operational classification [19].

### Molecular analysis of the SCN1A (rs2298771) polymorphism

We extracted genomic DNA from the whole venous blood using QIAamp DNA blood mini kits (provided by QIAGEN, USA, Catalog Number: 51104) and stored at − 20 °C till used. We measured the genomic DNA purity and concentration, from the controls and cases, using the NanoDrop™ 2000 Spectrophotometer (Thermo Scientific, Waltham, MA).

The genotyping of the *SCN1A (rs2298771)* polymorphism was carried out by the TaqMan single nucleotide polymorphism (SNP) Genotyping Assays (Applied Biosystem, Foster City, CA). The PCR primers and the variant type allele-specific TaqMan MGB probes were designed by Applied Biosystem. The SNP ID was C 11748767_20 for the *SCN1A (rs2298771)* polymorphism; and the chromosomal location was Chr.2:166036278.

The context sequence [VIC / FAM] was: TAGTCAAGATCTTTCCCAATTTCTG[C/T]TGTATGATTGGACATACAACTGTCT.

As a reporter at the 50 end of TaqMan MGB probe, VIC and FAM were used for the A-allele (Allele-1) and the G-allele (Allele-2), respectively.

We prepared 25 μL of the PCR reaction mix for qPCR analysis as follow: 7.25 μL DNase-free and RNase-free water, 12.5 μL TaqMan universal master mix II with UNG 2× (# 4440042, Applied Biosystem), 4 μL DNA template and 1.25 μL TaqMan assay 20×. We transferred PCR reaction mix to the 48-well reaction plate that was sealed using an appropriate cover. Then, centrifugation was done, and followed by loading into the step one real-time PCR (Applied Biosystem). The cycling stages of the reaction were as follow: stage I for UNG incubation for 30 s at 60 °C, stage II for polymerase activation for 10 min at 95 °C, stage III for PCR and included 40 cycles of denaturation at 95 °C for 15 s, annealing/extension at 60 °C for 60 s and finally stage IV for 30 s at 60 °C. We discriminated the alleles by asessing the fluorescence intensity at the endpoint.

**Fig. 1:**
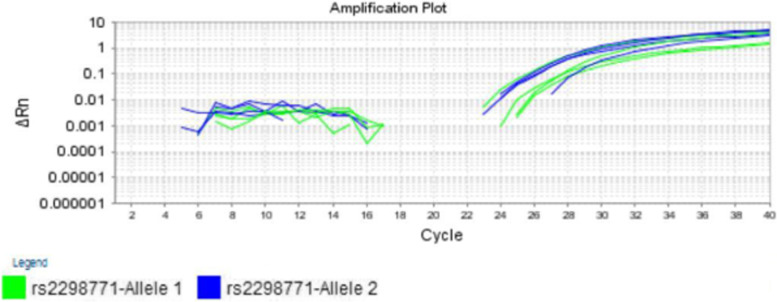
Real time PCR Amplification plots of the genomic DNA of four cases with the AG genotype of the *SCN1A-A3184G* polymorphism (Allele 1 = A-allele, Allele 2 = G-allele)

**Fig. 2:**
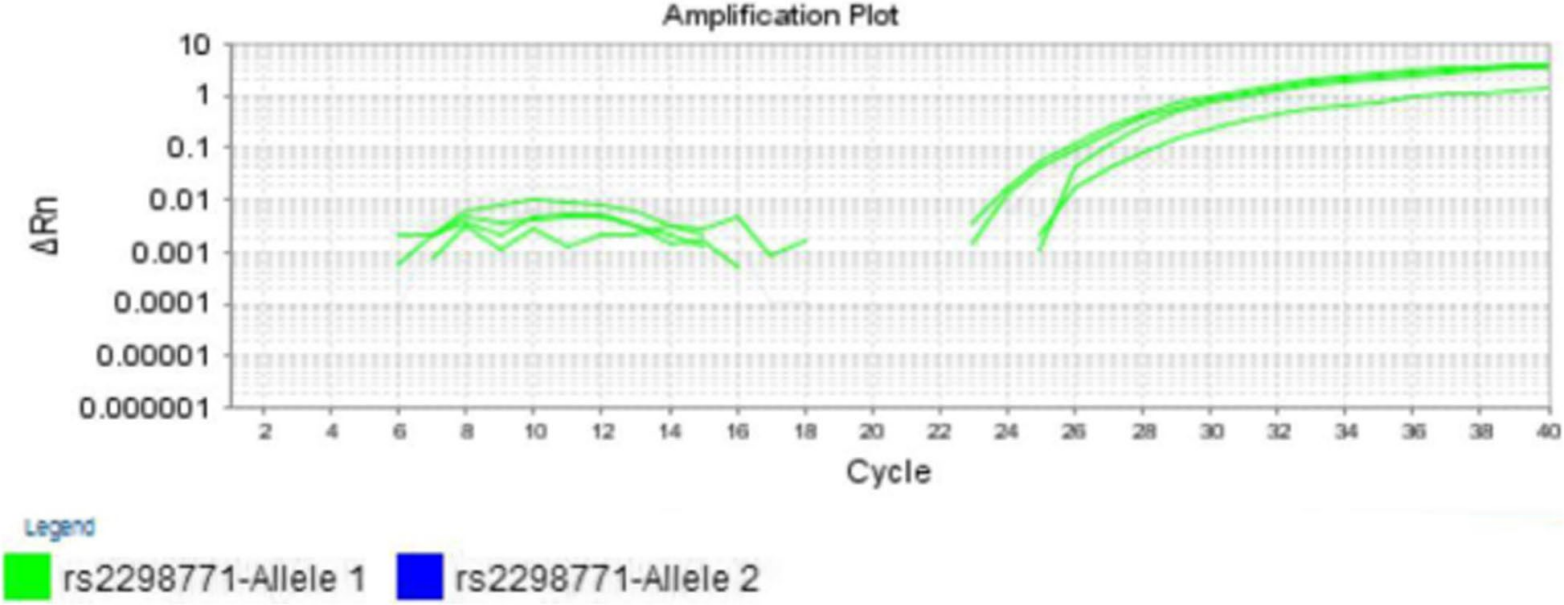
Real time PCR Amplification plots of the genomic DNA of four cases with the AA genotype of the *SCN1A-A3184G* polymorphism (Allele 1 = A-allele, Allele 2 = G-allele)

We analyzed the results of the measurements using the SDS software version 1.7 (Applied Biosystem), and detected the genotypes (Figs. 1 and 2). We randomly assayed 10% of the original specimens for replicate to exclude errors of genotyping. We did not detect discrepancies between the genotypes which were determined in duplicate.

### Statistical analysis

We analyzed data using the Statistical Package for the Social Sciences (SPSS) Version 25 (IBM Corp, Armonk, NY). Numbers (percent) were compared by Monto Carlo and chi-square tests (χ^2^). Parametric data (mean ± SD) were analyzed by the Student’s t-test while non-parametric data (median and interquartile range) were analyzed by the Mann–Whitney test. *P* values ≤0.05 were considered statistically significant. Genotypes and allelic frequencies of *SCN1A (rs2298771)* polymorphism were tested using the chi-square test to verify the agreement with Hardy–Weinberg equilibrium (*p* > 0.05 for each group).

## Results

### Descriptive data and characteristics of the study participants

Both AEDs resistant and AEDs responders groups were matched with the control group as regards the age, gender and body weight (*p* = 0.07, 0.347 and 0.462, respectively, Table 1). AEDs-resistant group showed higher seizers frequency, shorter time intervals since the last seizure attack and more abnormal EEG findings (*p* < 0.001, Table 2). No statistically significant differences existed between AEDs responders and resistant groups regarding the age of the first seizure attack (*p* = 0.642, Table 2). Regarding the type of seizures, the most frequent type of seizures in both groups was the tonic-clonic type that was detected in 63.8 and 76.1% in the AEDs responders and AEDs resistant groups, respectively. The other common types in the AEDs responder group were the tonic seizures (20.9%) and absence seizures (9.2%) while in the AED resistant group, absence seizures were detected in 10.4% and myoclonic seizures in 7.4% (Table 2).

**Table 1:** Characteristics of the study participants

	Control group (*n* = 163)	AEDs-resistant group (n = 163)	AEDs-responders (n = 163)	*p* value
Age (years)^a^	9.39 ± 2.27	9.44 ± 3.52	10.17 ± 3.98	0.07
Sex^b^				
Male	78(47.9)	70(42.9)	83(50.9)	0.347
Female	85(52.1)	93(57.1)	80(49.1)	
BW (Kg) ^a^	32.78 ± 10	31.46 ± 12.27	36.07 ± 17.93	0.462

Data were presented as mean ± SD ^a^ and numbers (percent) ^b^ and analyzed by Student’s t-test^a^ and chi-square test ^b^, respectively

*AEDs* Antiepileptic Drugs, *BW* Body Weight, *Kg* Kilogram, *N* Number, *P* Probability

**Table 2:** Seizure characters and antiepileptic therapies among cases with epilepsy

	AEDs resistant group (*n* = 163)	AEDs responders (*n* = 163)	*p* value
Types of seizures^a^			
Tonic-clonic	124(76.1)	104(63.8)	< 0.001^*^
Tonic	10(6.1)	34(20.9)	
Myoclonic	12(7.4)	0	
Clonic	0	10(6.1)	
Absence	17(10.4)	15(9.2)	
Electroencephalogram^a^			
Normal	33(20.2)	73(44.8)	< 0.001^*^
Generalized activity	130(79.8)	90(55.2)	
Antiepileptic drugs^a^			
Topiramate	12(7.4)	0	< 0.001^*^
Carbamazepine/ Oxcarbazepine	28(17.2)	44(27)	0.03^*^
Valproic acid	147(90.2)	134(82.2)	0.037^*^
Levetiracetam	68(41.7)	43(26.4)	0.003^*^
Polytherapy	86(59.7)	58(40.3)	0.002^*^
Monotherapy	77(42.3)	105(57.7)	
Duration since the last seizure attack (months)^b^	3(1–3)	14(12–24)	< 0.001^*^
Age of first seizure attack (years)^b^	3(0.75–6)	3(0.83–6)	0.642
Frequency of seizures per month^b^	2(1–4)	0.17(0.17–0.17)	0.001^*^

Data were expressed as numbers ^a^ (percent) and median ^b^ (interquartile range), and tested using chi-square ^a^ and Monto Carlo tests ^a^, and Mann-Whitney test ^b^

*AEDs* Antiepileptic Drugs, *N* Number, *P* Probability

^*^P ≤ 0.05 is significant

### The pattern of antiepileptic drugs among the patients

In the current study, valproic acid was the most frequently used AEDs in all groups (82.2% of the AEDs responder group and 90.2% of the AEDs resistant group) followed by levetiracetam in the AEDs resistant group (41.7%) and carbamazepine in the AEDs responder group (27%). There was a statistically significant difference between the both groups regarding the use of AEDs. Carbamazepine was more frequently used in the AEDs responder group (*p* = 0.03), while levetiracetam was more frequently used in the AEDs resistant group (*p* = 0.003). Topiramate was only used in the AEDs resistant group (Table 2).

### The distribution of SCN1A-A3184G genotypes and alleles among the study groups

The AG genotype of *SCN1A-A3184G* polymorphism was the predominant genotype in all groups (81.6, 76.1 and 73.6% for the control group, the AEDs responder group, and the AEDs resistant group, respectively, Table 3). No (GG) genotype was described in the studied populations. No significant differences existed between cases and controls regarding (AG) and (AA) genotypes and A- and G-alleles (*p* = 0.09 and 0.3, respectively).

**Table 3:** Distribution of the *SCN1A-A3184G* genotypes and alleles among the study groups

	Control group (*n* = 163)	AEDs-resistant (*n* = 163)	AEDs- responders (*n* = 163)	*p* values		OR (95%CI)
Genotypes (n)						
AG	133(81.6)	120(73.6)	124(76.1)	0.214	*p*_1_ = 0.08	1.59(0.94–2.69)
AA	30(18.4)	43(26.4)	39(23.9)		*p*_2_ = 0.223	1.39(0.816–2.38)
					*p*_3_ = 0.61	0.88(0.53–1.44)
Alleles (2n)						
A	193(59.2)	206(63.2)	202(61.9)	0.563	*p*_1_ = 0.296	0.845(0.617–1.16)
G (r)	133(40.8)	120(36.8)	124(38.04)		*p*_2_ = 0.471	0.891(0.651–1.22)
					*p*_3_ = 0.746	1.05(0.767–1.45)

Data were presented as numbers (percent) and analyzed by the chi-square test

*AEDs* Antiepileptic Drugs, *CI* Confidence Interval, *N* Number, *OR* Odds Ratio, *P* Probability, *P*_1_ for controls versus AEDs-resistant group, *P*_2_ for controls versus AEDs- responders, *P*_3_ for AEDs-resistant group versus AEDs- responders

We did not find significant differences between AEDs responders, AEDs resistant groups and the control group regarding the genotypes and alleles of the *SCN1A-A3184G* polymorphism (*p* = 0.214 for the genotypes and 0.563 for the alleles, Table 3). Moreover, we did not find significant differences between AEDs responders and resistant cases regarding the target genotypes and alleles (*p* = 0.61 and 0.746, respectively, Table 3).

### Associations between SCN1A-A3184G genotypes and characteristics of the patients

Regarding combined AEDs responders and resistant cases, Table 4 shows a higher seizure frequency and more generalized epileptiform EEG activity in the AG-genotyped cases (*p* = 0.004 and < 0.001, respectively). The frequent seizure types associating the AG genotype were tonic-clonic, tonic and absence seizures, while in patients with the AA genotype were tonic-clonic and tonic seizures. No significant differences existed between the AG and AA genotypes regarding AEDs, except for topiramate that was only used in cases with the AG genotype.

**Table 4:** Associations between the *SCN1A-A3184G* genotypes and characteristics of the patients

Characteristics	Epilepsy Cases		AEDs resistant group		AEDs responders		*p* value
Genotypes	AG(*n* = 244)	AA(*n* = 82)	AG(*n* = 120)	AA(*n* = 43)	AG(*n* = 124)	AA (*n* = 39)	
Family history^a^							*p*_1_ = 0.268
Negative	150(61.5)	56(68.3)	60(50)	32(74.4)	90(72.6)	24(61.5)	*p*_2_ = 0.006^*^
Positive	94(38.5)	26(31.7)	60(50)	11(25.6)	34(27.4)	15(38.5)	*p*_3_ = 0.19
Types of seizures^a^							
Tonic-clonic	176(72.1)	52(63.4)	97(80.8)	27(62.8)	79(63.7)	25(64.1)	*p*_1_ = 0.002^*^
Tonic	25(10.2)	19(23.2)	0	10(23.3)	25(20.2)	9(23.1)	*p*_2_ < 0.001^*^
Myoclonic	6(2.5)	6(7.3)	6(5)	6(14)	0	0	*p*_3_ = 0.262
Clonic	10(4.1)	0	0	0	10(8.1)	0	
Absence	27(11.1)	5(6.1)	17(14.2)	0	10(8.1)	5(12.8)	
EEG^a^							
Normal	60(24.6)	46(56.1)	11(9.1)	22(51.2)	49(39.5)	24(61.5)	*p*_1_ < 0.001^*^
Generalized epileptiform activity	184(75.4)	36(43.9)	109(90.9)	21(48.8)	75(60.5)	15(38.5)	*p*_2_ < 0.001^*^ *p*_3_ = 0.016^*^
AEDs ^a^							
Topiramate	12(4.9)	0	12(10)	0	0	0	*p*_1_ = 0.04^*^ *p*_2_ = 0.03^*^
Carbamazepine /Oxcarbazepine	50(20.5)	22(26.8)	11(9.2)	17(39.5)	39(31.5)	5(12.8)	*p*_1_ = 0.231 *p*_2_ = 0.03^*^ *p*_3_ = 0.02^*^
Levetiracetam	86(35.2)	25(30.5)	48(40)	20(46.5)	38(30.6)	5(12.8)	*p*_1_ = 0.432 *p*_2_ = 0.457 *p*_3_ = 0.028^*^
Valproic acid	215(88.1)	66(80.5)	115(95.8)	32(74.4)	100(80.6)	34(87.2)	*p*_1_ = 0.083 *p*_2_ < 0.001^*^ *p*_3_ = 0.352
Duration since the last seizure (months)^b^	3(0.75–5)	3(0.94–9)	2(1–6)	3(0.75–3)	18(12–24)	13(12–24)	*p*_1_ = 0.088 *p*_2_ = 0.029^*^ *p*_3_ = 0.139
Age of the first seizure attack (years)^b^	2(1–5)	1(0.33–3)	3(0.75–6)	3(1–9)	3(1–5)	2.5(0.67–8)	*p*_1_ = 0.015^*^ *p*_2_ = 0.03^*^ *p*_3_ = 0.229
Frequency of seizures (per month)^b^	13(12–24)	12(6–24)	2(1–5)	1(0.33–4)	0	0.17(0.17–0.17)	*p*_1_ = 0.004^*^ *p*_2_ = 0.05^*^

Data were expressed as numbers ^a^ (percent) and median ^b^ (interquartile range), and compared using the chi-square ^a^ and Monto Carlo tests ^a^, and Mann-Whitney test ^b^

*AEDs* Antiepileptic Drugs, *Epilepsy cases* AEDs responders plus resistant cases, *EEG* Electroencephalogram, *N* Number, *P* Probability, *P*_1_ for the AG versus AA genotypes in epilepsy cases, *P*_2_ for the AG versus AA genotypes in the AEDs-resistant group, *P*_3_ for the AG versus AA genotypes in AEDs-responders

^*^P ≤ 0.05 is significant

In the AEDs resistant cases, there were significant associations between the AG genotype and the earlier age of onset, the seizures frequency, the abnormal EEG activity, the positive family history of epilepsy, topiramate and valproic acid (*p* = 0.03, 0.05, < 0.001, 0.006, 0.03 and < 0.001 respectively, Table 4). While in AEDs responders, there were significant associations between the AG genotype and the abnormal EEG activity, levetiracetam and carbamazepine (*p* = 0.016, 0.028 and 0.02, respectively, Table 4).

## Discussion

Epilepsy is a highly heterogeneous disease with a confirmed genetic background [23, 24]. More than one quarter of the epilepsy-related genes encode ion channel proteins, including the ligand-gated ion channels (such as gamma-aminobutyric acid receptors, N-methyl-D-aspartate receptors and nicotinic acetylcholine receptors) and the voltage-gated channels (such as Ca^2+^, K^+^ and Na^+^ channels) [25]. The first *SCN1A* mutation was found in epilepsy patients by 2000 [23], but now many new *SCN1A* mutations have been identified making it the most common epilepsy-related gene [26]. In our preliminary research, we investigated the patterns and frequencies of *SCN1A-A3184G* alleles and genotypes among Egyptian children and adolescents with non-lesional epilepsy. We found that the AG genotype and the A allele are the predominant models among the study participants.

In the current study, we reported insignificant differences between epilepsy cases and the control group regarding the *SCN1A-A3184G* genotypes and alleles. No significant differences existed between AEDs responders and resistant cases regarding these genotypes and alleles. Lack of significant differences among our Egyptian patients could be explained on the basis of the ethnic variation. This was evidenced by Baum et al. in their large multi-ethnic study, where they considered the *SCN1A-A3184G (rs2298771)* polymorphism as the significant ethnic-related locus among symptomatic epilepsy patients (*p* < 0.001) while the frequencies of the other studied *SCN1A* loci *(rs10188577 and rs3812718)* did not differ according to the ethnicity [15]. However, the relatively small sample size could be a limiting factor in interpreting our results.

Our results also agree with Chou et al. who reported insignificant differences between 83 control subjects and 104 Taiwanese epileptic children regarding *SCN1A-A3184G* genotypes and alleles [14]. We also agree with Kang et al. who investigated 311 children for *SCN1A-A3184G (rs2298771)* and *SCN2A-G56A (rs17183814)* polymorphisms and tested their association with refractory seizures. They reported insignificant differences between controls and cases as regards the *SCN1A-A3184G* polymorphism, but the A allele of *SCN2A-G56A* polymorphism was associated with the refractory seizures [27]. Moreover, similar findings were described in a German study where *SCN1A-A3184G* polymorphism was not considered as a major contributor to the idiopathic generalized epilepsy [28]. We also agree with Hosseini et al. who evaluated the frequency of the *SCN1A (rs2298771 & 7,601,520)* and the *ABCB1 (rs1045642)* polymorphisms within the Iranian population with idiopathic refractory epilepsy. They enrolled 81 healthy subjects and 34 patients with idiopathic refractory epilepsy, and reported insignificant differences between the studied groups [29]. There is also agreement with another Chinese study including 471 patients with epilepsy (272 AEDs responders and 199 AEDs resistant cases). Authors studied the association of the responsiveness to AEDs with *SCN1A*, *SCN2A*, and *SCN3A* polymorphisms, and correlated any association with the mRNA expression of the neuronal sodium channels. They suggested an association between *SCN2A IVS7-32A > G* and the AEDs response, without any evidence of an effect on the mRNA expression or splicing. However, they reported a negative association between the *SCN1A-A3184G* polymorphism and the resistance to AEDs [30].

On the other hand, Lakhan et al. suggested that the AG genotype of the *SCN1A-A3184G* polymorphism was more frequent among North Indian epilepsy patients [*p* = 0.005; 95% confidence interval = 1.19–2.61, odds ratio = 1.76] [12]. In a meta-analysis study done by Li et al., the *SCN1A-A3184G (rs2298771)* polymorphism was significantly associated with the response to AEDs [31].

In the current study, we did not find the GG genotype of the *SCN1A-A3184G* polymorphism among the studied population. This is in contrast to a Slovenian study where the G allele was associated with a lower epilepsy risk and a high remission rate among patients with epilepsy [7]. These discrepancies could be mainly explained by the interethnic differences in the *SCN1A* alleles/genotypes distribution [20].

Interestingly, the current study suggested significant associations between the heterozygous AG genotype in the epilepsy cases with the higher seizure frequency and generalized epileptiform EEG activity. Possible associations were also suggested between the AG-genotyped patients with refractory epilepsy and the earlier age of onset of seizures and duration since the last seizure attack. On reviewing literature, no previous reports tested the associations between the *SCN1A-A3184G (rs2298771)* polymorphism and the predictors of refractory epilepsy. We would like to emphasize that the higher seizure frequency, generalized epileptiform EEG activity and the earlier age of onset of seizures were proved to be significant predictors of the AEDs response in previous studies [32, 33]. This observation could open the gate for considering the AG genotype of the *SCN1A-A3184G* polymorphism as a predictor for treatment response, and aid in designing the personalized medicine for epileptic children according to their genotype [34]. However, large scale multi-center studies are still needed to prove such associations.

Several studies were carried out to select the most appropriate AEDs in different types of epilepsy [9, 35]. The current study suggested that levetiracetam and carbamazepine were more frequently used in the AG-genotyped responders than those with the AA genotype. Regarding the AEDs resistant group, topiramate and valproic acid were more frequently used in children with the AG genotype while carbamazepine was more frequently used in the AA genotype. This observation highlights the possible role of the *SCN1A-A3184G* genotypes in the selection of the appropriate AEDs.

On reviewing literature, several studies were carried out to detect the possible pharmacological implications of the *SCN1A-A3184G* polymorphism in epilepsy management. A recent meta-analysis including eight studies reported that Asian patients with epilepsy and the GG genotype of the *SCN1A-A3184G* polymorphism are at a higher risk of carbamazepine resistance [36]. On the other hand, another meta-analysis including 18 studies (2546 patients) reported no association between the same polymorphism and the carbamazepine resistance [37]. Regarding the valproic acid, a Chinese study suggested that *SCN1A-A3184G* polymorphism has no effect on the drug response [38]. Actually, gathering data from different ethnic groups expand the knowledge about the genetic background of epilepsy and help in personalized medicine strategy.

### Study limitations

A single center study, the lack of serum AEDs levels assay and limited studied variables.

## Conclusions

No significant differences were found between Egyptian children and adolescents with non-lesional epilepsy and healthy controls regarding the frequency of *SCN1A-A3184G* polymorphism. We suggested significant associations between the AG genotype of the *SCN1A-A3184G* polymorphism and some predictors of refractory epilepsy. The *SCN1A-A3184G* genotypes might affect AEDs selection. Multicenter large-scale studies are still needed to validate our findings.

## Data Availability

The datasets generated during the current study are available in the Mendeley Data: 10.17632/mkjprydvst.1

## Abbreviations

AEDs: Antiepileptic Drugs
EEG: Electroencephalogram
PCR: Polymerase Chain Reaction
SCN: Sodium Channels
SCN1A: Sodium Channel, Alpha Subunit 1
SNP: Single Nucleotide Polymorphism
SPSS: Statistical Package for the Social Sciences
